# Multidisciplinary Conservative Treatment of Difficult Bile Duct Stones: A Real Alternative to Surgery

**DOI:** 10.1155/1997/42087

**Published:** 1997

**Authors:** E. Masci, L. Fanti, A. Mariani, S. Spagnolo, W. Zuliani, M. Castrucci, P. A. Testoni, A. Tittobello

**Affiliations:** 1 Department of Gastroenterology San Raffaele Hospital University of Milan Italy; 2 Department of Surgery San Raffaele Hospital University of Milan Italy; 3 Department of Radiology San Raffaele Hospital University of Milan Italy; 4 Department of Internal Medicine University of Milan Italy

## Abstract

56 patients with large CBD or intrahepatic stones underwent endoscopic and/or percutaneous treatment followed by extracorporeal shock wave lithotripsy. Percutaneous access to the biliary tract was chosen when an endoscopic approach was not possible (hepaticojejunostomy in 5 patients, 1 juxtapapillary diverticulum and I inflammatory bile duct stricture). Visualization of stones was achieved radiologically in 32 patients and by ultrasound in 24. The procedure was successful in 47 of 56 treated patients (83.9%). Clearance of the biliary tract was obtained in 25 cases (53%), whereas in 22 cases (47%) complete clearing of biliary tract was obtained only after endoscopic extraction of fragments (17 cases) or percutaneous (5 cases). The median number of shock waves in each session was 1725 (range 300–3166), which were applied during one (n=30), two (n=22) or three sessions (n=4). The only complications were 1 case of symptomatic hyperamylasemia and 3 cases of macrohematuria. In conclusion, extracorporeal lithotripsy combined with endoscopic and/or percutaneous treatment is a real alternative to surgery for difficult stones.


*HPB Surgery*, 1997, Vol. 10, pp. 229–234
Reprints available directly from the publisher
Photocopying permitted by license only

© 1997 OPA (Overseas Publishers Association)
Amsterdam B. V. Published in The Netherlands
by Harwood Academic Publishers
Printed in India

### Multidisciplinary Conservative Treatment of Difficult Bile Duct Stones: A Real Alternative to Surgery

E. MASCI[a], L. FANTI[a], A. MARIANI[a], S. SPAGNOLO[b], W. ZULIANI[b], M. CASTRUCCI[c], P. A. TESTONI[d] and A. TITTOBELLO[a]

[a]*Department of Gastroenterology,* [b]*Department of Surgery,* [c]*Department of Radiology, San Raffaele Hospital, University of Milan,* [d]*Department of Internal Medicine, University of Milan*

*(Received 7 June 1996)*

**56 patients with large CBD or intrahepatic stones underwent endoscopic and/or percutaneous treatment followed by extracorporeal shock wave lithotripsy. Percutaneous access to the biliary tract was chosen when an endoscopic approach was not possible (hepaticojejunostomy in 5 patients, 1 juxtapapillary diverticulum and 1 inflammatory bile duct stricture). Visualization of stones was achieved radiologically in 32 patients and by ultrasound in 24. The procedure was successful in 47 of 56 treated patients (83.9%). Clearance of the biliary tract was obtained in 25 cases (53%), whereas in 22 cases (47%) complete clearing of biliary tract was obtained only after endoscopic extraction of fragments (17 cases) or percutaneous (5 cases). The median number of shock waves in each session was 1725 (range 300–3166), which were applied during one (n=30), two (n=22) or three sessions (n=4). The only complications were 1 case of symptomatic hyperamylasemia and 3 cases of macrohematuria. In conclusion, extracorporeal lithotripsy combined with endoscopic and/or percutaneous treatment is a real alternative to surgery for difficult stones.**

*Keywords:* Bile duct stones, endoscopic papillosphincterotomy, extracorporeal shock-wave lithotripsy, percutaneous transhepatic cholangiography

### INTRODUCTION

Currently, the treatment of common bile duct (CBD) stones as well as both multiple and complex intrahepatic lithiasis, is no longer exclusively surgical. Both endoscopic and transhepatic percutaneous approaches, sometimes combined, may offer successful treatment for most cases [1–3]. However, when very large stones have to be managed or anatomic conditions interfere with their removal, the success rates of nonsurgical methods are much lower [4]. On the other hand, in the elderly and in cases with complicated stones, **surgery is marked by higher rates of morbidity and mortality, even though the figure has dramatically dropped in recent years [5–7].** Extracorporal lithotripsy has been demonstrated to be useful for treatment of large stones in the CBD and biliary intrahepatic ducts. Several studies confirming its effectiveness have been published [8–11]. We report here

Correspondence to: Enzo Masci MD Department of Gastroenterology San Raffaele Hospital via Olgettina 60 20132 Millan (ITALY)

our experience with the conservative treatment of large stones, both CBD and intrahepatic.

### METHODS

From 1989 to 1994, 56 patients, 30 men and 26 women, aged from 27 to 92 years **(median: 68 yr)**, underwent endoscopic and/or percutaneous treatment followed by extracorporeal shock-wave lithotripsy **the choice of non-surgical treatment was based on the old age of patients, on higher surgical risks factors and, for younger patients, because of previous multiple surgical intervention.** 44 patients were treated because of evidence for large CBD stones not amenable to routine endoscopic measures (Dormia Basket and mechanical lithotripsy). 12 patients had intrahepatic lithiasis, 3 of them with associated CBD stones. **The maximum stone size of CBD stones per patient ranged from 11.5 to 70 mm (median: 25 mm) and of intrahepatic stones from 10 to 35 mm (median: 17.5).** In 3 of 56 patients, there was massive lithiasis of the intra- and/or extrahepatic biliary tract.

For 7 patients, percutaneous access to the biliary tract was chosen because endoscopic access was not possible for anatomical reasons (hepaticojejunostomy in 5 patients) or by conditions that might limit endoscopic sphincterotomy (1 juxtapapillary diverticulum, 1 inflammatory bile duct stricture) in these cases **the maximum stone size per patient ranged from 6.5 to 10 mm (median: 10).** Following endoscopic treatment and lithotripsy, 2 patients underwent percutaneous extraction of intrahepatic fragments (Tab. I).

Stones and biliary system were visualized by contrast medium injection through an endoscopically placed naso-biliary drainage catheter in 25 patients, or through transhepatically placed catheters in 7 patients. All these patients were treated with the first generation Dornier HM3 lithotripter, with the patient in the supine position partially immersed in water. In 24 other patients the stones were visualized by ultrasound in 13 cases, before 1990, by the Wolf Piezolith 2300 lithotripter (WP) and in 11 subsequent cases, by the MPL 9000 lithotripter. During the treatment with Dornier HM3 (32 cases), patients underwent analgesia with Fentanyl in doses varying from 0.05 to 0.15 mg, combined with 5 to 10 mg of Diazepam whenever necessary, whereas in the remaining cases no analgesia was needed. If at the end of the session there was no evidence of fragmentation, patients underwent an additional session. Fragmentation was verified the day after the lithotripsy was performed, after a series of washings through the drain positioned in the biliary tract. The choice between endoscopic removal of fragments and percutaneous re-treatment was made only after the extent of fragmentation clearly indicated they could be removed and there was no evidence of unassisted elimination. Complications and side effects following both endoscopic procedures and lithotripsy were recorded in order to have an overall complication rate for the combined treatment.

TABLE I Procedures Employed before Extracorporeal Lithotripsy and Biliary Stone Location

| | CBD | ID | CBD+ID |
|---|---|---|---|
| P T C (*n*=7) | 7 | 0 | 0 |
| Endoscopy (*n*=47) | 37 | 9 | 1 |
| Combined (*n*=2) | 0 | 0 | 2 |
| Total (*n*=56) | 44 | 9 | 3 |

PTC: Percutaneous transhepatic ducts
CBD: Common bile duct
ID: Intrahepatic duct

### RESULTS

56 patients underwent a total of 86 treatments (average 1.5). Only a single session was required in 30 cases (7 WP, 8 MPL, 15 Dornier), a second session in 22 cases (3 WP, 2 MPL, 17 Dornier), a third session in only 4 cases (1 MPL, 3 WP). The median of the number of shock waves in each session was 1725 (range: 300–3166). 3 obese patients, 2 others with CBD stones, and one with intrahepatic stones were not treated because of the impossibility of correctly positioning the stones in the shock-wave focus, either radiologic or ultrasonographic. Stones fragmentation was achieved in 51/56 patients (91%). In 47 of 56 treated patients (83.9%), the procedure was successful. Cholangiographic controls at the end of treatment demonstrated un-assisted clearance of the biliary tract in 28/47 cases (59.5%), whereas in 19/47 cases (40.5%) complete clearing of the biliary tract was obtained by endoscopic extraction of fragments (17 cases) or percutaneously (2 cases), with the Dormia basket or balloon (Fig. 1). Among the 12 patients treated for intrahepatic stones, either alone (9 cases) or combined with CBD stones (3 cases), the methods failed for only one subject with a 20 mm stone, treated with Dornier HM3. The reason for this failure was intolerance to the procedure. In 2 of the remaining 8 patients with CBD stones, failure was due to lack of fragmentation. Both patients, in fact, had bulky CBD stones (35 and 40 mm) and were treated with the WP 2300 and the MPL 9000. 3 patients, two of them treated with the Dornier HM3 and the other with the MPL 9000, did have fragmentation of the stones, but the massive lithiasis and the overload of material prevented complete clearing of the biliary tract. For another 3 subjects, the sessions were interrupted due to intolerance to the procedure (2 Dornier HM3, 1 MPL 9000). The results after treatment with the different lithotripters are summarized in Table II.

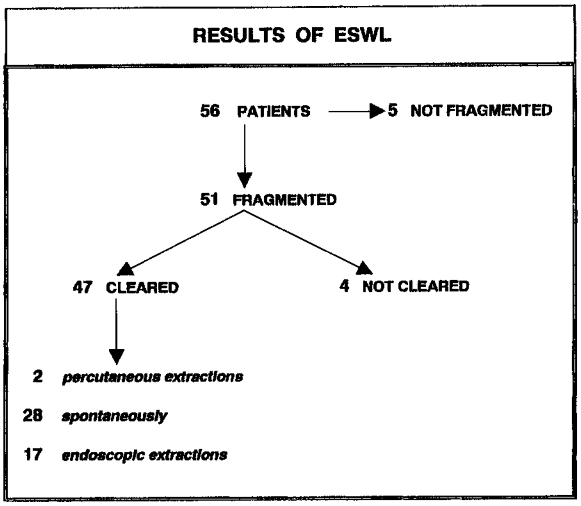

FIGURE 1

There were no serious complications with either the endoscopic or the radiological procedures. One case of symptomatic transitory hyperamylasemia and three cases of macrohematuria, that resolved spontaneously (1 MPL, 2 Dornier) were observed after treatment.

### DISCUSSION

The surgical approach for biliary stones was the standard method until about 10–15 years ago. Its results have been excellent, with low rates of both morbidity and mortality [12,7]. With the advent of endoscopic sphincterotomy, surgical exploration of the biliary tract has also lost its place as priority treatment for young patients without high surgical risks. The development of laparoscopic cholecystectomy as one of the mini-invasive surgical techniques, has additionally stimulated the use of interventional endoscopy for removal of stones from the biliary tract. However, in about 10% of cases, endoscopic treatment may fail due to the impossibility of fragmentation and removal of bulky stones or even of stones in the intrahepatic biliary tract. In addition, some anatomic features may limit the endoscopic removal of stones or even make endoscopic treatment difficult, if not impossible [4].

In cases in which the standard endoscopic procedure is ineffective, several techniques have been combined with it to improve the approach to "difficult" stones of the biliary tract. In fact, the use of chemical solvents has been introduced, but this technique has been shown to be inconvenient, time-consuming, and not totally free of side-effects. In 40% of cases, in fact, patients complained of nausea, vomiting and

TABLE II Number and Rate of Clearance, Fragmentation and Failure after Treatment with Different Lithotripters

| | Clearance | | Fragment | | Failure | |
|---|---|---|---|---|---|---|
| | N | (%) | N | (%) | N | (%) |
| TOTAL | 47/56 | (83,9) | 51/56 | (91,0) | 9/56 | (16,1) |
| WP | 12/13 | (92,3) | 12/13 | (92,3) | 1/13 | (7,6) |
| MPL | 8/11 | (72,7) | 9/11 | (81,8) | 3/11 | (27,2) |
| D | 27/32 | (84,3) | 30/32 | (93,7) | 5/32 | (15,6) |

WP: Wolf Piezolith 2300 Extracorporeal Piezoelectric Lithotripter (EPL)
MPL: Dornier MPL 9000 Shock Wave Lithotripter
D: Dornier HM3 with Waterbath diarrhea, so that the successful outcome was less than 50% [13,14]. Mechanical lithotripsy may also be ineffective if the stone is larger than 25 mm [4]. When the endoscopic approach to the biliary tract was difficult or even impossible, a multidisciplinary approach together with transhepatic treatment combined with extracorporal lithotripsy with shock-waves was, in our experience, of the utmost usefulness. In fact, in our series we have observed stone fragmentation in 91% of our cases. Endoscopic and/or radiologic treatment combined with extracorporeal lithotripsy enabled us to completly clear the biliary tract in 83.9% of cases. The only 3 cases in which it was impossible to focus the stone represent the evident limitations of the first generation equipment designed for the treatment of renal stones that we employed. It is probable that updated machines, supplied with a radiographic targetting system, would have enabled us to treat all the patients in our series.

In any case, our results are in accordance with data in the literature [10,15–17]. In our experience, in accord with a recent work of Lindstrom *et al.* [16], the 32 patients treated with Dornier HM3 lithotripter did not need any general anesthesia and for all cases Diazepam plus Fentanyl was effective. For two patients for whom general anesthesia was not possible, treatment had to be stopped. Although Sauerbruch *et al.* [15] reported the need for general anesthesia in 75% of cases, in our series it was not required and this not only simplifies the procedure but also reduces the risks for older patients while permitting good tolerance to the procedure. The clearing rates of the biliary tract achieved with the multidisciplinary approach to difficult stones, are comparable to the results of surgery, for which the rate of residual stones in such cases is approximately 5 to 10% [18]. Recently, a prospective randomized trial [19] comparing extracorporeal with intracorporeal electrohydraulic lithotripsy (EHL) showed no differences in stone-free rates between the two therapies. The conclusion of the Investigators however, was that large and multiple stones are probably best treated with extracorporeal lithotripsy. In these cases, in fact, for complete duct clearance, up to three additional endoscopic interventions may be necessary after EHL and extracorporeal lithotripsy is better accepted by the patient than an endoscopic session.

In our series, overall complications, spontaneously resolved in all cases, were 7.1%. There was no case of cholangitis, particularly dangerous in this kind of pathology, probably thanks to the drainage placed in the biliary tract in almost all cases and to antibiotic treatment during the process of clearing. In our series, there was no evidence of major complications, as has, instead, been described by some other groups [20–22]. Currently, through a combination of endoscopy, radiologic intervention and extracorporeal shock-wave lithotripsy, we have a real possibility of succesfully treating most of the difficult stones of the biliary tract. Extracorporal lithotripsy also provides a real alternative to surgery for difficult stones.

**Even though the results of our study do not enable us to say definitely whether conservative treatment is favorable in terms of cost-benefit, the costs in our institution for extracorporeal lithotripsy, about 590 US $/session, and for sphincterotomy, about 780 US $, are comparable with the cost of surgical exploration of CBD (1017 US $). Considering the low rate of complication with conservative treatment and the higher morbidity of surgery, and the need sometimes for more complex interventions (bilio-digestive anastomosis, liver resection), the cost of non-surgical treatment is not very different than that of surgery and, therefore, cost may not be, in our opinion, a criterion of selection.**

**In conclusion, the choice between surgical and non-invasive removal of stones should in our point of view be made individually for each patient, taking into consideration the risk for surgery for that patient but also, in patients without surgical risk, the fact that for their biliary patology they had already been submitted to previous multiple surgical interventions. The presence of very large stones (>35 mm), the only ones not cleared in our experience, could militate against non-surgical approach.**

### *Acknowledgements*

The Authors wish to thank Mr. Gaetano Lattaruolo for his collaboration in performing our procedures.

### *References*

[1] Cotton, P.B. (1984). Endoscopic management of bile duct stones (apples and oranges). *Gut*, **25**, 587–97.

[2] Kaway, K., Akasaka, Y. and Murakamy, K. (1974). Endoscopic sphincterotomy of the Ampulla of Vater. *Gastrointestinal Endoscopy*, **20**, 148–51.

[3] Venbrux, A.L. (1992). Interventional Radiology in the biliary tract. *Current Opinion in Radiology*, 4(3), 83–92.

[4] Sivak, M.V. (1989). Endoscopic management of bile duct stones. *American Journal of Surgery*, **158**, 228–38.

**[5] Escarce, J.J., Shea, J.A., Chen, W., Qian, M.A. and Schwartz, J.S. (1995). Outcomes of open cholecystectomy in the elderly: a longitudinal analysis of 21.000 cases inthe prelaparoscopic era. *Surgery*, 117, 156–164.**

[6] Tytgat, G.N.J., Huibregtse, K., Bartelsman, F.W.M. and Schneider, B. (1987). La sphincterotomie endoscopique. Revue general. *Acta Endoscopica*, **17**, 51–60.

**[7] Moreaux, J. (1995). Traditional surgical bile duct stones: a prospective study during a 20-year experience. *American Journal of Surgery*, 169, 220–226.**

[8] Sauerbruch, T., Delius, M., Holl, J., Wess, O., Hepp, W., Brendel, W. and Paumgartner, G. (1985). Fragmentation of gallstones in humans by extracorporeal shock wave treatment. Hepatology, **5**, 977.

[9] Sauerbruch, T., Holl, J., Sackmann, M., Jocham, D., Delius, M., Brendel, W. and Paumgartner G. (1987). Treatment of bile duct stones by extracorporeal shock waves. *Seminary Ultrasound CTMR*, **8**, 155–61.

[10] Sauerbruch, T. and Stern, M. The Study Group for Shock-Wave Lithotripsy of Bile Duct Stones (1989). Fragmentation of bile duct stones by extracorporeal shock waves: a new approach to biliary calculi after failure of routine endoscopic measures. *Gastroenterology*, **96**, 146–52.

[11] Martin, L.G. Ambrose, S.S., Elias, D.L. and Amerson, J.R. (1988). Extracorporeal shock wave lithotripsy of intrahepatic stones: case presentation and review of the literature. *American Surgery*, **54**, 311–14.

[12] Mc Sherry, C.K. (1989). Cholecystectomy: the gold standard. *American Journal of Surgery*, **158**, 174–78.

[13] Murray, W.R. Laferla, G. and Fullarton, G.M. (1985). Choledocho-lithiasis in vivo stone dissolution using methyl tertiary butylether. *New England Journal of Medicine*, **312**, 217–20.

[14] Neoptolemos, J.P. Hoffman, A.F. and Moossa, A.R. (1986). Chemical treatment of stones in the biliary tree. *British Journal of Surgery*, **73**, 515–24.

[15] Sauerbruch, T., Holl, J., Sackmann, M. and Paumgartner, G. (1992). Fragmentation of bile duct stones by extracorporeal shock-wave lithotripsy: a five-year experience. *Hepatology*, **15**, 208–14.

[16] Lindstrom, E., Borch, K., Kullman, E.P., Tiselius, H.G. and Inse, I. (1992). Extracorporeal shock wave institution experience. *Gut*, **33**, 1416–20.

[17] Dobrilla, G., de Pretis, G., Felder, M., Amplatz, S., Chilovi, F. Piazzi, L. Comberlato, M., Benvenuti, S. and Vallaperta, P. (1992). Extracorporeal shock-wave lithotripsy in bile duct stones refractory to papillosphincterotomy. *European Journal of Gastroenterology and Hepatology*, **4**, 475–479.

[18] Neoptolemos, J.P., Carr-Locke, D.L. and Fossard D.P. (1987). Prospective randomised study of preoperative endoscopic sphincterotomy versus surgery alone for common bile duct stones. *British Medical Journal*, **294**, 470–474.

[19] Adamek, H.E., Buttmann, A., Wessbecher, R., Kohler, B. and Riemann, J.F. (1995). Clinical comparison of extracorporeal piezoelectric Lithotripsy (EHL) in difficult bile duct stones. A prospective randomized trial. *Digestive Diseases and Sciences*, **40**(6), 1185–92.

[20] Sauerbruch, T. and Stern, M. (1989). Fragmentation of bile duct stones by extracorporeal shock waves. *Gastroenterology*, **96**, 146–152.

[21] Staritz, M., Rambow, A. and Grosse, A. *et al.* (1990). ESWL for fragmentation of extra and intra-hepatic bile duct stones: indications, success and problems during a 15 months clinical experience. *Gut*, **31**, 222–225.

[22] Wenzel, H. Greiner, L. and Jakobeit, C.H. *et al.* (1989). Extrakorporale Stosswellenlithotripsie von Gallengangssteinen. *Deutsche medizinische Wochenschrift*, **114**, 738–743.

### COMMENTARY - J TOOULI

The management of common bile duct stones has changed significantly in the last 10 to 15 years. Currently, there is little need to explore the bile duct via an open approach in order to treat stones in the duct. The impact of the newer approaches has been reduced morbidity and early recovery for patients. The newer non-operative techniques have not always been subjected to fair or valid comparative studies with open surgery and they have been introduced at a time when open surgery also has become much safer. Then whilst there may be little significant difference in mortality between the open and minimally invasive techniques, there undoubtedly is a better morbidity in favour of the latter approach.

The success rate of the minimal access techniques is high, however for patients with very large stones or patients who have had multiple operations in the upper abdomen, the success rate is reduced. In this study the treatment of 56 patients with difficult large bile duct stones is reported. The authors have used extracorporeal shock wave lithotripsy (ESWL) to assist in their management of these patients via endoscopic and percutaneous approaches. The results clearly indicate the appropriateness and success of this approach as most experienced hepatobiliary surgeons would recognise that these are the most difficult of patients with choledocholithiasis to treat.

The only draw back of the technique described in this paper is the requirement of access to an extracorporeal lithotripsy device. These are expensive devices which cannot be justified economically on their management of bile duct calculi alone. Hence, the approach described in this report can only be used by clinicians who might have access to ESWL that is generally used for the treatment of renal calculi.

### *References*

Seminars in Laparoscopic Surgery: Bile Duct Calculi Guest Editor Toouli, J. Volume 2, Number 2, June 1995 WB Saunders Company.

Prof. J Toouli
Head of Gastrointestinal Surgical Unit
Department of Surgery
Flinders Medical Centre.

